# Y Choromosomal Microdeletion Screening in The Workup of Male Infertility and Its Current Status in India

**Published:** 2013-12-22

**Authors:** Ramaswamy Suganthi, Vijayabhavanath Vijayakumaran Vijesh, Nambiar Vandana, Jahangir Fathima Ali Benazir

**Affiliations:** 1 Department of Biotechnology, Dr.G.R. Damodaran College of Science, Coimbatore, India; 2 Molecular Biophysics Unit, Indian Institute of Science, Banglore, India

**Keywords:** Male Infertility, Y Chromosome Microdeletions, Intracytoplasmic Sperm Injection, Sequence-Tagged Site

## Abstract

Spermatogenesis is an essential stage in human male gamete development, which is regulated by many Y chromosome specific genes. Most of these genes are centred in a specific region located on the long arm of the human Y chromosome known as the azoospermia factor region (AZF). Deletion events are common in Y chromosome because of its peculiar structural organization. Astonishingly, among the several known genetic causes of male infertility, Y chromosomal microdeletions emerged as the most frequent structural chromosome anomaly associated with the quantitative reduction of sperm. The development of assisted reproductive techniques (ART) like intra-cytoplasmic sperm injection (ICSI) and testicular sperm extraction (TESE) helps to bypass the natural barriers of fertilization, but it increases the concern about the transmission of genetic defects. Experimental evidence suggested that the men with Y chromosomal microdeletions vertically transmitted their deletion as well as related fertility disorders to their offspring via these ART techniques. In India, infertility is on alarming rise. ART centres have opened up in virtually every state but still most of the infertility centres in India do not choose to perform Y chromosomal microdeletion diagnosis because of some advanced theoretical reasons. Moreover, there is no consensus among the clinicians about the diagnosis and management of Y chromosomal microdeletion defects. The current review discusses thoroughly the role of Y chromosome microdeletion screening in the workup of male infertility, its significance as a diagnostic test, novel approaches for screening Y deletions and finally a systematic review on the current status of Y chromosome microdeletion deletion screening in India.

## Introduction

Infertility is a major public health problem with significant social, psychological and economic impact. In world literature, there is a paucity of accurate study to estimate the actual prevalence and incidence of infertility all around the globe. Noticeably a study carried out by Boivin et al. (1) estimated that the prevalence of infertility ranged from 3.5 to 16.7% with an overall median prevalence of 9% where more than half of them seek medical care. Worldwide, the incidence of infertility among the general population is estimated to be about 10-15% (2).

Infertility is defined as the diminished or absent ability to conceive or produce an offspring after at least one year of unprotected sexual intercourse (3). Being a parent and having a family is the primary vision of most people in their adulthood. When it ends in infertility, it brings in enormous emotional trauma, feelings of sadness, depression and anger. Infertility is indeed a very painful struggle. Traditionally, women in infertile couples bear the sole responsibility for the failure to conceive. However, in reality, infertility is not just limited to women alone. It is identified that about 50% of infertility are of male origin (4). In male factor infertility, the human Y chromosome plays a pivotal role by regulating the male germ cell development and maintenance (5). The research on Y specific candidate genes and their relationship with idiopathic male infertility has been under scrutiny for several decades now, but sadly, the research is still at its infant stages and continues to pose vital challenges for researchers and clinicians alike, but the picture appears to have become even more challenging with reports from several groups around the world, that there are probably several genes or gene sequences within the target region of the Y chromosome which are deleted. Furthermore, recent studies have shown that a significant proportion of men with severe idiopathic infertility have microdeletions in the Y chromosome (6). The rapid advancement in assisted reproductive techniques gives hope to millions of infertile couples to have their own baby at the same time it raises serious concerns about the vertical transmission of genetic defects, including Y chromosomal microdeletions as well as related fertility disorders to their sons (7). It implies the necessity for screening Y chromosomal microdeletions as a routine diagnostic test in the workup of male infertility.

In India, infertility is on an alarming rise. It opens an attractive new market for fertility business. Infertility clinics with assisted reproduction technology (ART) facilities have opened in virtually every state, both in rural and urban areas but the quality varies considerably. Even after several reports emanating from expert groups in India and abroad about the possibility of transmission of undetected molecular genetic defects to the intra-cytoplasmic sperm injection (ICSI) born babies, half of the centres do not choose to perform the advanced genetic screening analysis like Y chromosomal microdeletion diagnosis for varying reasons. Denying the benefits of advanced genetic screening techniques to the ART born babies will adversely affect their normal and reproductive health. In this review, we will systematically outline the role of Y chromosome microdeletion screening in the workup of male infertility, its significance as a diagnostic test, novel approaches for screening Y deletions, pros and cons of currently available techniques and finally to discuss critically about the current status of Y chromosomal microdeletion analysis in India.

### Genetic aetiology of male infertility

Crossing all the barriers, male infertility is on a rise than ever before. In about 25-30% of cases the possible reason for the causes of male infertility is unknown, and the condition is termed as idiopathic male infertility. It is believed that genetic factors play the key role in the aetiology of this condition (8). On analysis of the semen, the nature of the abnormality can be identified (Table 1) (9). Today the advances made in research regarding the molecular and cellular mechanism of spermatogenesis helps to characterize many disorders previously considered as idiopathic. The most important of them are hypogonadotrophic hypogonadism, mutations in the androgen receptor, cystic fibrosis transmembrane conductance regulator gene mutation, genetic polymorphisms and Y chromosome linked infertility. Among these, the chromosomal abnormalities are found much more frequently in infertile men, with an incidence of 4-16% as compared to an incidence of 0.4% in the fertile population (10).

**Table 1:** Nomenclature for common semen variables proposed by WHO [94]


Medical term	Condition

**Normozoospermia**	Normal ejaculate with normal values of semen variables
**Azoospermia**	Absence of sperm in the ejaculate
**Aspermia**	Fail to ejaculate semen
**Oligozoospermia**	Sperm concentration less than 20x10^6^/ml
**Asthenozoospermia**	Fewer than 50% of sperm have low motility.
**Teratozoospermia**	Fewer than 30% sperm with normal morphology
**Hematospermia.**	Semen containing red blood cells (RBC)
**Pyospermia **	Semen containing White blood cells (WBC)
**Polyzoospermia **	Too high concentration of sperm


### The human Y chromosome

The human Y chromosome consists of a short (Yp) and a long (Yq) arm. Cytogenetic analysis helped to identify several different Y regions; the pseudo autosomal portion (including PAR1 and PAR2), the euchromatic and heterochromatic regions. The two pseudo autosomal regions (PAR1 and PAR2) located on both telomeric ends cover around 5% of the chromosome. They are identical with the appropriate telomeric segments of X chromosome and the genes localized in PARs (PAR1 has 14 genes, and PAR2 has 3 genes) show an autosomal pattern of inheritance (11). Euchromatin regions consist of Yp and the proximal part of Yq corresponding to Yq11 with a size of 24 megabases (Mb), while the distal part of the longarm Yq12 is made of a genetically inert region called heterochromatin which may vary in length in different male population. From the evolutionary point of view, the sex chromosomes (X and Y chromosomes in the human genome) evolve from a homologous pair of autosomes (12). Suppression of recombination between nascent sex chromosomes endorsed them to evolve independently; it in turn resulted in the accumulation of male beneficial genes for the sex determination and spermatogenesis in a specific region of Y chromosome called male-specific region of the Y chromosome (MSY) previously called non-recombining region of Y (NRY) (11). Instead of the usual recombination "intra chromosomal gene conversion" or non-reciprocal transfer of genetic information occurring between duplicated sequences within the chromosome take place (13).

### Genomic organisation of the male-specific region of the Y chromosome

The MSY comprises 95% of the chromosome’s length. The euchromatic sequences of the MSY belong to three discrete classes; X-transposed, Xdegenerate and ampliconic. Together these three sequences constitute around 23 Mb of the chromosome, including 8 Mb on the short arm (Yp) and 14.5 Mb on the long arm (Yq, 14). The X-transposed region (combined length of 3.4 Mb) shows 99% similarity to the X chromosome. This can be acquired through the process of X to Y transposition that occurred about 3-4 million years ago (15). X-degenerate sequences (8.6 Mb) represent the single copy gene or pseudogene homologues of X-linked genes that appear to be the remnants of ancient autosomes from which the sex chromosomes co-evolved. Ampliconic sequences constitute the major portion of the MSY euchromatic sequence, where sequence pairs show greater than 99.9% identity organized in massive palindromes. These amplicons are distributed in seven blocks on Yp and Yq and whose combined length is 10.2 Mb. The most outstanding features of the ampliconic regions of Yq are the presence of eight massive palindromes. It collectively comprises 5.7 Mb, or one-quarter of the MSY euchromatin and six out of eight carrying recognized protein-coding genes, all of which seem to be expressed specifically in the testes (14). Among the 156 transcriptional units located in the euchromatin sequence of MSY, 78 were protein-coding units encoding at least 27 distinct proteins or protein families. The X-degenerate sequences encode 16 distinct proteins, which were expressed ubiquitously except the sex determining SRY, which is found to be expressed predominantly in the testes. The ampliconic sequences encode 9 of the MSYs 27 distinct proteins which are specifically expressed in testis indicating the importance of ampliconic sequence in Y chromosomal architecture and thereby regulating spermatogenesis. Ampliconic sequences exhibit the highest density of genes as well as the lowest density of interspersed repeat elements. These sequences comprise sixty coding genes organised in nine MSY specific protein-coding gene families and 75 non-coding transcription units; 65 are members of 15 MSY-specific families and the remaining 10 occur in single copy (14, 16).

### Mapping in male-specific region of the Y chromosome

In 1976, Tiepolo and Zuffardi (17) made the first attempts at mapping the Y chromosome and thereby proposed a hypothesis that "a gene or gene cluster on the long arm (Yq11) is essential for fertility". After a decade, the first molecular map based on the development of linear deletion interval maps using Y-specific DNA probes was developed which subdivided the Y chromosome into seven deletion intervals (18). Genetic mapping is impossible in MSY due to the suppression of meiotic recombination. Therefore, Y chromosome is the more appropriate target for physical mapping. Physical maps are based on collections of overlapping ribosomal DNA (rDNA) clones covering an entire genome. But the early efforts to construct accurate, high-resolution physical maps of the MSY were found to be tedious because of mere availability of specific DNA markers, presence of large-sized intra-chromosomal repetitive sequences, or amplicons (19). By using about 200 Y specific sequence tagged sites (STS’s) Vollrath et al. (20) constructed a 43 interval deletion map of human Y chromosome, which refined the seven interval map of Vergnaud et al. (18). These arrays of STS have been extensively used to build scaffolds of overlapping recombinant DNA clones. As a result, the complete physical map of human Y chromosome was generated with 196 overlapping DNA clones, which covered 98% of the euchromatic region (19). This in turn helped with the sequencing and mapping of MSY using BAC clones (21). The availability of an MSY reference sequence has led to the expansion of the current knowledge of spermatogenesis and Y chromosomal infertility (14).

### Y chromosomal microdeletions

Y chromosome microdeletions (YCM) represent the absence of DNA segments or gene(s) from the functionally active part of the Y chromosome. The first insight into the correlation between the Y chromosomal microdeletion and male infertility came from the studies of Tiepolo and Zuffardi in 1976 (17). Later on with the development of STS and YAC based mapping, several interstitial microdeletions which are present on the long arm of Y chromosome (Yq11) were identified (19-21). Currently, the Y chromosomal microdeletions assigned to be the most frequent structural chromosomal anomaly associated with failure in sperm production with an overall frequency of 1 to 58%, specifically 15-20% of idiopathic azoospermic men, 7-10% of idiopathic oligozoospermic men and 2-3% of the candidates for ICSI are carriers of microdeletions (22). The frequency variation among various studies is mainly due to the lack of proper patient selection criteria, ethnic variation among the study population and differences in the experimental designs (23).

### Azoospermia factor

**Fig 1:**
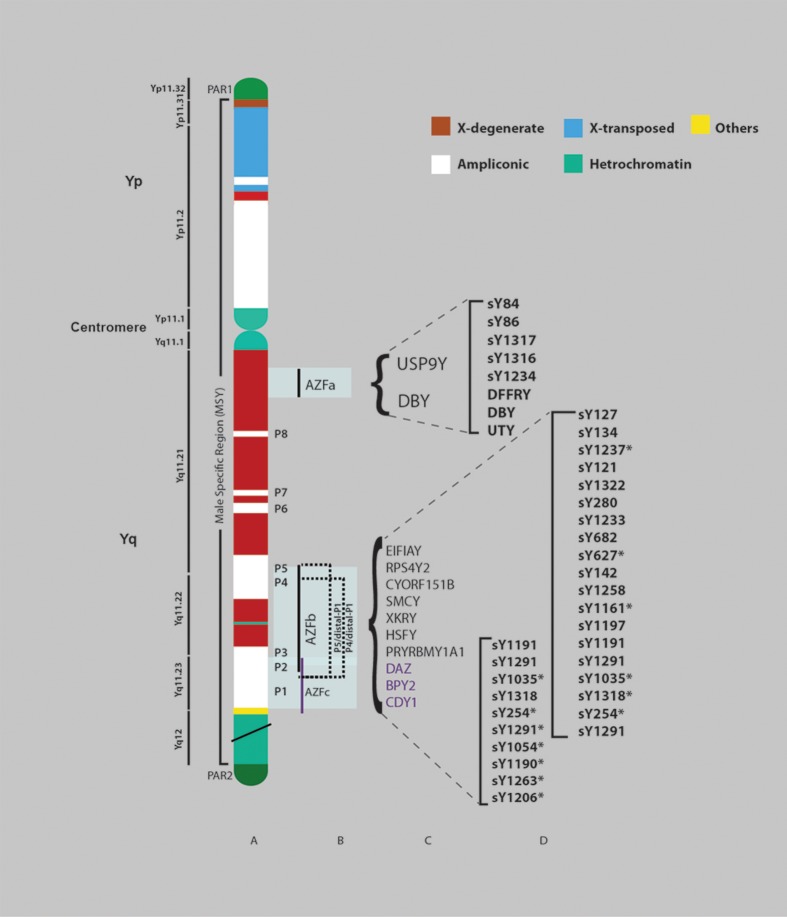
Diagram of the human Y chromosome showing AZF deletions. A. Schematic representation of the structure of human Y chromosome showing pseudoautosomal region (PAR1, PAR2) , centromere and male-specific region of the Y chromosome (MSY) with eight palindromes (P1-P8), heterochromatic sequences and three classes of euchromatic sequences: X-transposed, X-degenerate and ampliconic. B. Schematic map of common AZF deletions with corresponding candidate genes. C. STS markers associated with AZFa, b and c regions respectively, used for routine screening analyses of Y chromosomal microdeletions globally (*; Multiple copy).

Besides the factors which control testicular differentiation and maturation, on the Y chromosome, a third genetic factor or gene cluster located on the distal portion of the long arm of the Y chromosome (Yq11 or deletion interval 5 and 6) controlling the spermatogenesis is termed as the azoospermia factor (17). It is said to be the hotspot region of Y chromosomal microdeletion screening analysis. Detailed molecular analysis subdivided the azoospermia factor into three sub-regions, AZFa, AZFb, AZFc along with a fourth recently proposed AZFd region (24, 25). Each of these regions comprises functionally active genes and transcription units related to spermatogenesis. To date, 14 proteins coding genes and two pseudogenes are found in the AZF locus (26). Partial or complete deletion of AZF regions impairs spermatogenesis. Several clinically relevant microdeletion patterns have been identified in the AZF locus, namely AZFa, P5-proximal P1 (AZFb), P5-distalP1 (AZFbc), P4-distal P1 (AZFbc) and b2/b4 (AZFc) (27). The deletions are caused by the intrachromosomal recombination between homologous sequences (14). The AZF region and the deletion patterns with corresponding STS markers are schematically represented in figure 1.

### AZFa locus

The AZFa locus lies in the proximal region of the Yq 11 (D3-D6) or in the deletion, subinterval 5C of the human Y chromosome (25, 28). It is about 800 kb long and contains two functional single copy genes, USP9Y and DBY and at least 11 pseudo genes. USP9Y (Ubiquitin specific peptidase 9, Y-linked), which is comprised of 46 exons and spans 159 kb of genomic DNA whereas, DBY (Dead box on Y) contains 17 exons and a length of 16 kb (29). USP9Y gene belongs to a member of the peptidase C19 family which encodes a protein which acts like ubiquitin C-terminal hydrolase and is expressed ubiquitously. The basic function of USP9Y gene is to increase the efficiency of spermatogenesis like a "fine-tuner" (30-32). DBY gene codes for ATP dependent RNA helicases in humans which play a significant role in pre-meiotic spermatogonia phase. It has a characteristic DEAD (Asp-Glu-Ala-Asp) box at the sequence level and the proteins encoded by the DBY gene are expressed only in testis tissue (33). Moreover, mutation events in the candidate genes may lead to infertility (29, 31).

### Mechanism of AZFa deletions

AZFa deletions are low frequency microdeletions resulting from homologous intrachromosomal recombination between two human endogenous retroviral sequences HERV15yq1 and HERV15yq2 located in the proximal Yq11. The complete AZFa deletion removes ~792 kb of the DNA sequences, including the two candidate genes (34).

### Diagnosis of AZFa deletions

Microdeletions in the azoospermia region are common mutational events. They can be detected using polymerase chain reaction (PCR) with the help of STS. The most frequently used gene specific, single copy STS markers to detect and study extension of AZFa deletions are DFFRY, DBY, sY83,sY85, sY86, sY84, sY87, sY88 sY90, sY1317, sY1316 and sY1234 (25, 35-38).

### Clinical significance

The partial removal of AZFa region has been associated with hypo-spermatogenesis whereas the complete deletion of AZFa region blocks the production and maturation of germ cells in the seminiferous tubule. Consequently, the testicular biopsy shows the Sertoli cell-only (SCO) phenotype. It is virtually impossible to retrieve mature sperm upon testicular sperm extraction (TESE) for the use in IVF/ICSI (39). Transmission of AZFa deletion to the offspring is also reported (29).

### AZFb locus

AZFb is located in the middle region of Yq11 or at the deletion interval between P5 palindrome and the proximal arm of P1 palindrome of the MSY. It overlaps with the AZFc region by 1.5 Mb and it spans around 6.2 to 7.7 Mb of MSY sequences (13). After the identification of the first AZF candidate gene, RBMY, several single copy genes and multicopy gene families were observed, some of them located on the AZFb locus itself and others which share the AZFc region. The single copy protein coding genes include EIF1AY (eukaryotic translation initiation factor 1A, Y-linked), RPS4Y2 (Ribosomal protein S4 Y isoform 2), CYORF15A (chromosome Y open reading frame 15A), CYORF15B (chromosome Y open reading frame 15B) and SMCY (Smcy homologue, Y chromosome). Additionally "seven multicopy gene families namely XKRY (XK, Kell blood group complex subunit-related, Y-linked), HSFY (Heat shock transcription Factor Y), RBMY1A1 (RNA binding motif protein, Y-linked, family 1, member A1), PRY (PTPN13-like, Y linked), CDY (Chromodomain Y), BPY2 (Basic protein Y 2) and DAZ (deleted in azoospermia)" are present in the AZFb locus (40) and all the members of these gene families show a testis specific pattern of expression (41).

### Mechanism of AZFb deletions

The homologous recombination between the Palindrome P5 and the proximal arm of palindrome P1 of the Yq results in complete AZFb deletions. Complete P5/proximal-P1 (AZFb) deletion takes out 6.23 Mb spanning 32 genes and all the members of the testis-specific gene families present in the AZFb locus (13).

### Diagnosis of AZFb deletions

STS markers sY127 and sY134 are frequently used for the primary detection of AZFb deletion (16). For the extension analysis, sY117, sY114, sY1015, sY135, sY143, sY142, sY145, sY127,sY134, sY1237, sY121, sY1322, sY280, sY1233, sY682, sY627, sY142, sY1258, sY1161, sY1197, sY1191, sY1291, sY1035, sY1318, sY254 and sY1291 markers are used (36, 38). Gene specific markers in this region are EIF1AY, PRY, TTY2 and RBM1 (42).

### Clinical significance

Detection of the AZFb deletion has both diagnostic and prognostic value. Deletions in AZFb region leads to pre-meiotic spermatogenic arrest or SCOS and finally results in azoospermia. It is impossible to recover mature sperm upon TESE (41, 43).

### AZFbc deletions

Deletions which extend from P5 to the distal arm of P1 (P5/distal-P1 deletions) and from P4 to the distal arm of P1 (P4/distal-P1 deletions) together constitute the AZFbc deletions. (Earlier it was thought to be AZFb+c deletions). AZFbc deletions are considered to be a significant class of recurrent deletions in Y-chromosome also representing the largest of the deletions in the human genome (13). A deletion in P5/distal-P1 region takes up to 7.66 Mb, including 42 genes or transcripts whereas, P4/distal-P1 deletion removes 7.03 Mb along with 38 gene copies.

Non-homologous recombination between P5/ distal-P1 or between P4/distal-P1 explains the underlying mechanism behind AZFbc deletions (13). On the contrary, one study reported homologous recombination mechanism for P5/distal-P1 deletions. The same group identified seven different types of deletions within AZFb and AZFc regions. Most of them show non homologous mode of recombination (44). AZFbc deletions resulted in impaired spermatogenesis and have no chance of sperm retrieval through TESE (39).

### AZFc locus

The AZFc region is one of the most exhaustively studied AZF locus associated with male infertility. AZFc locus is mapped to the distal part of the Yq or deletion subintervals 6C-6E (25). The AZFc sequence spans about 4.5 Mb and comprises of six distinct families of amplicons including six large inverted repeats and three large direct repeats. Out of the six inverted repeats three of them are massive palindromes (P1, P2 and P3) with a size of 4.0 Mb (45). The AZFc locus contains 21 candidate genes and 11 families of transcription units specifically expressed in testis. Among the 11 families, 7 families including AZFc-exclusive sequence families, GOLGA2LY1 (Golgi autoantigen, golginare subfamily a, 2-like, Y-linked 1) and CSPG4LYP1 (chondroitin sulfate proteoglycan 4-like, Y-linked pseudogene 1) are specifically located in the AZFc deletion interval. The important candidate genes in this deletion interval are four copies of the DAZ (deleted in azoospermia), three copies of BPY2 (basic protein on Y chromosome 2) and two copies of CDY1 (CDY1a and CDY1b; chromodomain protein, Y chromosome 1) gene family (45, 46). The first identified and well-studied AZFc gene was DAZ. All the members of DAZ gene family encode RNA binding proteins, probably involved in the regulation of mRNA translation, thereby serving as a vital gene for spermatogenesis (47).

### Mechanism of AZFc deletions

AZFc deletions denote the most frequent type of deletion pattern observed among azoospermic and severe oligozoospermic patients. The homologous recombination between sub-amplicons b2 and b4 in palindromes P3 and P1 cause AZFc deletions. The bordering of the AZFc with the highly repetitive heterochromatic region of Yq12 may also trigger a high percentage of unequal intra-chromosomal recombination during meiotic stage of spermatogenesis which may increase the chance of AZFc deletion. The estimated sizes of AZFc deletions are around 3.5 Mb and result in the elimination of 21 genes along with seven families of transcription units, which are solely located within the deleted region (45).

### Diagnosis of AZFc deletions

The STS markers sY254 and sY255, specific for DAZ gene, are used for the initial screening of AZFc deletions (47). Extension of the deletion can be identified using a large set of STS markers including sY1192, sY1191, sY1291, sY 1035, sY1318, sY254, sY1291, sY1054, sY1190, sY1263, sY1206 sY 602 (BPY2), sY 1198, sY579, sY1125 and sY639 (CDY1) (38, 45).

### Clinical significance

The patients with AZFc deletions show capricious phenotypical features, which range from azoospermia to mild/severe oligozoospermia, and it can apparently be transmitted to the male offspring, hence the screening of AZFc deletions has a diagnostic and preventive value. The variations in the phenotype observed may be due to the genetic background of the patient screened, exposure to the environmental factors, size of deletion or 'progression of spermatogenesis failure with time' (28). Sperm retrieval from testicular tissue is possible in AZFc-deleted men when going for ICSI (48).

### Partial AZFc deletions and gr/gr deletions

The peculiar structural organization of AZFc region makes it more prone to large scale structural rearrangements (45). Recent reports indicate that several partial AZFc deletions occur in the AZFc region as a result of recombination between the sub-amplicons located within the AZFc locus. Among these gr/gr, b1/b3 and b2/b3 were identified to be clinically relevant for male infertility (49). The gr/gr deletions excise 1.6 Mb of the AZFc region, including four copies of the DAZ gene and one of three copies of the BPY2 gene. Since vertical transmission is observed, the gr/gr deletion likely reduces the fertility of the male offspring (50). In some men with gr/gr deletions, subsequent gene duplication helps to restore the gene copy number (51). The b2/b3 and b1/b3 deletions also reduce the copy number of the AZFc candidate genes and alter the normal spermatogenesis (52). The genotype-phenotype correlation, incidence and clinical relevance of AZFc partial deletions are not yet clear.

### Genetic screening methods for the diagnosis of Y chromosomal microdeletions

#### Novel approaches for screening Y chromosomal microdeletions

The rapid growth of the molecular diagnostics helps to introduce a wide array of molecular techniques to detect the smaller interstitial deletions in the Y chromosome. Suspension array technology (SAT) is one among this kind. SAT is based on flow cytometry principles. It simultaneously analyses hundreds of molecular targets during a single reaction. In this technique, the oligonucleotide probes are allowed to hybridize with microsphere beads of unique fluorescent label. Once the hybridization is over the probe hybridized microspheres are examined using the suspension array analyser. SAT has been increasingly used to detect the Y chromosomal microdeletions among the infertile patients (53). The technique is rapid, specific, sensitive and cost effective. However, it possesses a few disadvantages like comparatively low array size, problems in hybridization and difficulties in optimizing a single specific annealing temperature for the entire experiment. Array-comparative genomic hybridization (CGH) is another powerful molecular tool used for analysing sub microscopic Y deletions (54, 55). Another approach for screening Y deletions is the "use of the capillary electrophoresis technique combined with fluorescent multiplex PCR" (56).

#### The sequence tagged site- polymerase chain reaction method

The STS-PCR technique is considered to be the gold-standard method for the laboratory diagnosis of Y chromosomal microdeletions. In the STSPCR technique, a DNA sample would be tested for the presence of STS based on polymerase chain reaction. The procedure involves many automated cycles of DNA synthesis in a standard laboratory thermocycler. Afterwards, the PCR products are detected with the help of agarose gel electrophoresis. The presence of the amplified DNA bands indicates the existence of the known target sequence and vice versa. The deletion may be a gene or gene family or may be an unknown sequence depending upon the STS used (42, 57). While conducting PCR-based deletion analysis, several questions have puzzled the mind of clinicians.

#### Why PCR-based deletion analysis is routinely used for detecting the Y chromosomal microdeletions? What factors should be considered while performing it?

In the early days of research, karyotyping was commonly used to identify the macrodeletions in the long arm of Y chromosome. Since the conventional cytogenetic testing fail to detect the smaller interstitial deletions, southern blotting took up the charge to demonstrate the macro as well as the microdeletions that might be associated in azoo/oligozoospermia (18). Since karyotyping and southern blotting are labour-intensive, time consuming, costly and complex techniques, most laboratories have switched to PCR which is a simple, reliable,reproducible, less time consuming, cost effective, sensitive and easily automated technique allowing multiplexing. Once the PCR based deletion map was established (20) the search for the interstitial deletions of the Y chromosome based on PCR markers began (58), and soon it got accelerated and hundreds of papers were published within two decades. The major factors which influence the PCR based deletion analysis are as follows.

#### Selection of the polymerase chain reaction markers

The availability of the nucleotide sequence of the MSY (14) made it possible to select the best PCR markers from a pool of STS markers (38). When choosing markers, several factors must be considered: The finest and informative markers for the PCR based deletion analysis are single copy markers or the markers limited to a small region of the Y chromosome. The gene markers or repetitive markers like multi copy clustered or dispersed markers show negative results only when a large portion of the Y chromosome is deleted, and the presence of the PCR amplification products will not indicate all the copies of the target region are present. Hence the multi copy markers are less informative (35). The human Y chromosome, as a hotspot of mutational events, displays structural polymorphism based on ethnic background and geographical histories which will in turn reflect on the PCR markers (35, 59). Therefore, careful selection of highly specific, non-polymorphic (markers which are present in fertile men and absent in infertile men) STS markers is needed to detect the clinically relevant microdeletion patterns (16, 24).

#### Origin of the deletions and selection of DNA samples for screening

Y chromosomal deletions are either inherited through paternal germ line (7) or occur as de-novo events. Most of the Y deletion cases are of de novo origin (7, 28). Most likely, the event takes place in the pre-fertilization stages, although deletions could also be a post-fertilization event (60). If a Y-deleted sperm fuses with an egg, it gives rise to a Y-deleted child. On the other hand, if the deletion occurs as a post fertilization event, it will give rise to mosaicism (normal Y chromosome in leukocytes and deleted Y chromosome in sperm or testicular DNA). So the basic question arises at this point is which DNA sample should be screened for the detection of Y deletions in the patients opting for ART? A vast number of studies reported the incidence of Y chromosomal microdeletions in the lymphocyte DNA (6, 14, 25). Some of the studies observed a similar deletion frequency in lymphocyte and testicular DNA (61, 62). On contrary few studies found a weak germ cell mosaicism in oligozoospermic patients (63-65). Unfortunately these studies have not reported the extent and origin of the deletions. In one of the studies, evaluating the relation between sperm DNA damage and leukocyte DNA integrity, DNA integrity, cleavage rate and embryo quality were positively correlated with leucocyte DNA integrity (66). Furthermore, in azoospermic patients there will be no sperm in the ejaculate and testicular biopsy collection is still a highly invasive technique. Also, the collection, processing and the protocol standardization of sperm DNA isolation and PCR amplification are much more complex than the lymphocyte DNA. Considering all these facts, lymphocyte DNA is the cheapest and readily available sample for basic Y chromosomal microdeletion screening.

#### Polymerase chain reaction quality control

PCR amplification failure leads to the false interpretation of results. The use of high-quality DNA, appropriate internal and external positive and negative controls reduce the false negatives. Moreover, the European Academy of Andrology (EAA) guideline for PCR setup and internal quality control is currently of high value (16).

#### Reliability of STS markers

STS are short known DNA sequences whose location in the genome is mapped. The concept of STS was first put forward by Olson et al. (67). STS offers high speed, convenient, reliable and low cost genetic screening analysis. Today about 1287 Y-specific STS, including 992 single-copy and 285 multi-copy STS have been generated and mapped to MSY (38). It was proven that there is no correlation between the frequency of microdeletions detected and the number of STS analysed (68). However, for the multiplex STS-based PCR microdeletion analysis the primary screening which includes two sets of STS markers in each AZF sub-region is able to detect over 95% of the deletions (16). Once deletion has been detected, secondary screening should be done using 20-30 STS to detect the extent of deletions (69). If multiple discontinuous deletions are observed the result should be verified by southern blotting (70).

#### Patient selection for Y chromosomal microdeletion screening

Y chromosomal microdeletions are frequently associated with the quantitative decrease in the sperm production (27) and also coexist with other male infertility disorders, testicular cancer and other forms of human malignancies (71, 72). Furthermore, the Y deletions are inherited by the offspring; therefore, all the patients who were the candidates of assisted reproductive techniques should be also screened for Y chromosomal microdeletions. If Y chromosomal microdeletions are observed, subsequent genetic counselling should be provided to the affected couples.

#### Growing genetic concerns of assisted reproductive technologies

The development of ART began with the successful application of in vitro fertilization (IVF) in 1978. The standard IVF technique was not so effective for the treatment of people with severe sperm defects. The introduction of ICSI, the technique by which an egg is fertilized with the injection of a single sperm greatly accelerated the practice of ART globally (73). Since the ICSI technique bypasses all the natural mechanisms and filters related to normal fertilization, it raises serious concern about the transmission of known and unknown molecular genetic defects to the offspring. Now, the evidence from various studies shows an increased risk of congenital malformations and chromosomal aberrations in children born through ICSI when compared to the general population. The major risks include multiple gestations, low birth weight, premature birth, higher illness morbidity, hearing defects, genitourinary defects, imprinting defects and chromosomal aberrations (74-80). Recent reports have shown an increased risk of gene mutations in the ART offspring, irrespective of genetic background (78). It can be speculated that, the occurrence of cellular damage in the egg during ICSI procedure increases the incidence of de-novo chromosomal abnormalities in the developing embryo (81). In addition, the transmission of Y chromosomal microdeletions, CFTR gene mutations and DNA repair defects may possibly affect the health of child born after ICSI (77, 81). Therefore, proper genetic testing and counselling should be undertaken to reduce the genetic risk associated with ART.

#### Genetic counselling

Genetic counselling is the art of communication between a professional counsellor and a patient about a genetic disorder (82). Genetic counselling is mandatory and now a reality in almost all IVF centres. Today we have a fair knowledge about the adverse effects caused by Y chromosomal microdeletions, and it is also proven to be a potential genetic disorder. Therefore, genetic counselling should be compulsory to prevent propagation of this fearful disorder. In one study, it is reported that most of the couples choose IVF or ICSI using either the sperm of the partner or donor sperm only after given proper genetic counselling about their Yq deletions. In some cases, the couples choose to select female embryos for transfer (83). Pre-implantation genetic diagnosis (PGD) seems to be a potential alternative strategy for the couples dealing with Yq microdeletions (84).

#### Y chromosomal screening analysis in India

India, the second most populated country in the world, exhibits enormous diversity in terms of language, culture and ethnicity. According to the provisional reports released on March 31, 2011, the Indian population has increased to 1.21 billion with a decadal growth of 17.64%, and a total fertility rate (TFR) of 2.8 children born per woman (85). About 15-20% of married couples belongs to sub- or infertile category and a small fraction of these couples opt for ART in India (86). In the last two decades, several studies have reported the incidence of Y chromosomal microdeletions in the Indian population (Table 2) and emphasised on the need for the molecular diagnosis of deletions in the workup of male infertility (87-92). Even though a large number of infertility clinics are present in India, half of them still rely on classic cytogenetic analysis to find out genetic defects. Most of the infertility centres in India do not choose to perform Y chromosomal microdeletion diagnosis because of some advanced theoretical reasons including the test having no significance in the management of the infertility, doubts regarding the credibility of diagnostic techniques, lack of information on genetic counselling and the variation in the frequencies. Whatever the reasons, not testing the Y chromosome deletions is likely to increase the prevalence of complex genetic diseases associated with Y chromosomal rearrangements and will deleteriously affect the reproductive health of the patients and their family.

**Table 2:** Summary of the literatures on Y chromosomal microdeletion analysis in Indian population


Reference	Region Of Study	Sample Studied	No Of Infertile Men Screened	Control		No Of Sts Markers Used	Frequency Of Deletions			
				Fertile men	Normozoospermic men		Azoo	Oligo	Others	Total

**Ambasudhan et al., 2003**	Varanasi	Blood/Testis biopsy	177	?	?	29	8(5.6)%	1(3.3%)	0	9(5%)
**Dada et al., 2003**	New Delhi	Blood	83	25	0	6	7(9.58%)	1(10%)	0	8(9.6%)
**Thangaraj et al., 2003**	Kolkata	Blood	340	230	0	37	29(8.5%)	0	0	29(8.5%)
**Athalye et al., 2004**	Mumbai	Blood	100	5	0	18	8(29.63%)	0	4(5.48%)	12(12%)
**Swarna et al., 2004**	Hyderabad	Blood	70	?	?	5	4(44.4%)	5(55.5%)	0	9(12.8%)
**Rao et al., 2004**	South India	Blood	251	?	?	24	5(1.99%)	4(1.59%)	1(0.39%)	10(3.98%)
**Dada et al.,2004**	New Delhi	Blood	133	50	0	8	7(?)	1(?)	0	8(6.01%)
**Dada et al., 2006**	New Delhi	Blood	140	50	0	8	?(?)	?(?)	0	8(5.7%)
**Mitra et al., 2006**	New Delhi	Blood/Semen	14	13	0	19	4 (?)	1 (?)	0	4(28.6%)
**Viswambaran et al.,2007**	Tamilnadu	Blood	30	20	0	6	? (?)	? (?)	?	4(13.3%)
**Saktivel et al., 2008**	Tamilnadu	Blood/Semen	147	0	140	34	0	5(7.24%)	14(18.18)	19(12.9%)
**Abid et al., 2008**	Mumbai	Blood	200	50	0	8	3(3%)	0	3(3%)	6(3%)
**Mitra et al., 2008**	New Delhi	Blood	170	0	101	19	9(5.29%)	0	0	9(5.29%)
**Suganthi et al., 2009**	Tamilnadu	Blood	215	?	?	12	? (7.4%)	? (3.7%)	0	24(11.1%)
**Pandey et al., 2010**	Varanasi	Blood	64	?	?	5	? (?)	?(?)	0	3.33%
**Suganthi et al., 2011**	Tamilnadu	Blood	100	25	?	12	12(34.29%)	10(25%)	0	22 (29.3%)


#### The frequency of AZF deletions in the Indian population

The frequency of Y chromosome microdeletions in the Indian population ranges from 3 to 29.34 % (87- 93) with an average frequency of 8.1%. It is speculated that the variation in the frequency of Y deletions is mainly due to the ethnic background and study protocol. In one study, a total of 340 azoospermic Indian men was analysed of which 8.5% showed Y chromosome deletions, in which AZFc deletion was the most common (82.8%), followed by AZFb (55.2%) and AZFa (24.1%, 94). Another study reported the frequency of Yq microdeletions in the Indian population as 9.63% (89). A multiplex PCR assay for 18 loci of the Y-chromosome performed on infertile Indian men showed that 12% of the patients carry microdeletions, and the most commonly detected loci were DYS240 and DY6219 (90). Ali et al. (91) have ascertained so far a total of 109 cases with male infertility from Bangalore and have shown deletions exist at a frequency of 5.5% in the AZFc region only. In a study by Hellani et al. (95), a total of 257 patients with idiopathic oligo or azoospermia were screened for Y-chromosome microdeletion by typing 19 STS markers in AZF regions. Among these, six patients had deletions in the AZFc region. One case had a deletion in both AZFa and AZFc regions. In another study on Indian males, a total of 215 azoospermic infertile men were tested for the presence of 12 STS markers using multiplex PCR. The observed frequency of deletion was about 11.1%, among them the azoospermic men showed a higher frequency of deletions (7.4%) than the severe oligozoospermic men (3.7%) (92) [The summary of the studies on Y chromosomal microdeletion analysis in the Indian population is shown in table 2]. As the ethnic and environmental backgrounds affect the structural arrangements of the Y chromosome, it is necessary to choose the STS markers carefully for screening Y chromosome microdeletions based on ethnic background.

## Conclusion

In the era of assisted reproductive techniques, particularly relating to ICSI, the study of Y chromosomal microdeletions helps to open up new horizons. We now have the technology to test for Y chromosome microdeletions and have improved knowledge regarding who should be tested for Yq microdeletions. Additionally, the Y chromosomal deletion tests have a precise diagnostic, prognostic and preventive value. Once a deletion is observed in an infertile man it helps the clinicians to avoid empirical and often expensive treatments to improve fertility, and it also gives information about the chance of finding sperm in the testes of azoospermic men and about sperm cryopreservation in oligozoospermic men. Serious ethical issues may arise if clinicians promote the desire of couples for a child without considering the risks involved. In future, huge demand arises for developing new molecular technologies as well as standardized protocols which give reliable results and also help to increase efficiency, decrease cost and technical difficulty of the procedure. These advancements allow a more widespread use of Y chromosome microdeletion screening test in infertility clinics and andrology labs.
